# Validity of Airway Ultrasound in Correlation with Cormack-Lehane Grading in Obese Patients: A Cross-Sectional Study

**DOI:** 10.5812/aapm-142701

**Published:** 2024-03-24

**Authors:** Ahmed El-Tawansy, Ahmed Mohamed Salama Elnajar, Hossam Abdel Baky Mahmoud, Mohamed Ibrahim Amin, Ahmed Abd Elmohsen Bedewy

**Affiliations:** 1 Department of Anesthesia and Surgical Intensive Care, Helwan University, Cairo, Egypt; 2 Department of Anaesthesia and Surgical Intensive Care, Faculty of Medicine, Zagazig University, Zagazig, Egypt; 3 Department of Radiology, Faculty of Medicine, Zagazig University, Zagazig, Egypt

**Keywords:** Ultrasound, Laryngoscopy, Obesity

## Abstract

**Background:**

Ultrasound (US) of the upper airway has the potential to be a valuable addition to traditional clinical evaluation methods.

**Objectives:**

This work aimed to assess the validity of US in correlation with Cormack-Lehane grading (CLG) in obese patients.

**Methods:**

This cross-sectional work was performed on 78 patients ranging in age between 21 and 60 years, both genders with the American Society of Anesthesiologists (ASA) II-III individuals and body mass index (BMI) 30 kg/m² or more, under general anesthesia with endotracheal tube placement. Each separate finding by the US and conventional clinical airway assessment methods before anesthesia induction correlated to the CLG of the same patient after the induction of anesthesia. Grades III and IV are categorized as difficult laryngoscopy.

**Results:**

A significant positive association existed among CLG and duration of US measures, pre-epiglottis spaces (Pre-E) ratios, to the distance between a point mid away vocal cords and epiglottis, Pre-E, ratio of hyomental distance extension/hyomental distance neutral and Mallampati; however, there was a significant negative correlation with skin to anterior commissure, hyomental distance extension, hyomental distance neutral, sternomental distance, and thyromental distance (P < 0.05). The ratio between Pre-E over the distance between the epiglottis and a point midway through the vocal cords at cut-off > 2.23 can discriminate difficult laryngoscopy with sensitivity 100% and specificity 100% and area under the curve of 1.

**Conclusions:**

The sonographic assessment of the upper airway aids in predicting individuals who might have challenges with airway management. A reliable indicator of a challenging laryngoscopy was the sonographic parameter ratio of Pre-E to the distance between the vocal cords' midway point and the epiglottis.

## 1. Background

The term "difficult airway" does not have a universally agreed definition, although it refers to a combination of many factors related to the management of the airway (1). The categorization includes challenging installation of the supraglottic airway (SGA), challenging laryngoscopy, challenging ventilation using a mask or SGA, and challenging or unsuccessful endotracheal intubation. The Cormack-Lehane Grade (CLG) is widely accepted as a benchmark for classifying challenging laryngoscopy in anesthetic studies (2-4).

Several risk variables have been discovered to predict challenging airways. The factors considered in this study are demographic factors (e.g., age, gender, and race), prior occurrence of obstructive sleep apnea (OSA), body mass index (BMI), anomalies of the upper teeth, capacity for moving the lower teeth in the forefront of the upper teeth, inter-incisor gap, adjusted Mallampati score, thyromental distance, and capacity for extension and flexion of the cervical spine, in addition to the circumference of the neck (5).

Clinical screening examinations are generally impractical in emergency and critical care situations due to the frequent presence of confused, sluggish, unwilling, and disoriented patients who are unable to comply with instructions or assume proper positions. Efforts continue in the quest for a straightforward and non-intrusive method that would provide a more precise evaluation of the patient's airway (6, 7).

## 2. Objectives

The purpose of this work was to evaluate the validity of the US with regard to correlation to CLG in obese patients.

## 3. Methods

This cross-sectional work was performed on 78 patients.

(a) Inclusion Criteria: The age range between 21 and 60 years old, both genders with the American Society of Anesthesiologists (ASA) II-III individuals and BMI 30 to < 30 kg/m², under laparoscopic procedures, bariatric procedures, and any procedure necessitating general anesthesia with endotracheal tube insertion. 

The research was conducted under the authorization of the Institutional Review Board of Zagazig University Hospitals, Egypt. The participants provided informed written permission.

(b) Non-inclusion criteria: Individual refusal, airway anatomical deformities caused by tumors or masses, individuals with thyroid swellings (goiter), and airway pathologies, such as edema, burning, and arthritis. 

(c) Exclusion criteria: Patients required rapid sequence intubation, any change in anesthesia method, scheduled fiberoptic intubation, and uncooperative patients.

Each participant had been exposed to history taking, clinical assessment, neck X-ray lateral view to exclude any abnormalities that might interfere with the technique, and laboratory tests (complete blood count [CBC], liver and renal functioning tests, and coagulation profile).

**Figure 1.**
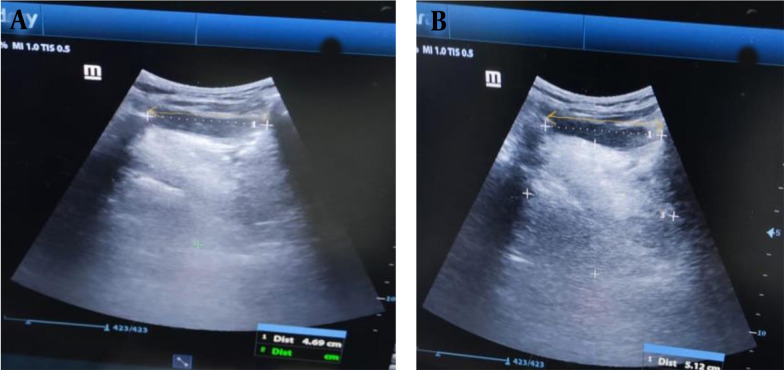
The hyomental distance of the patient in A, a neutral position and B, a fully extended neck (yellow arrow).

**Figure 2.**
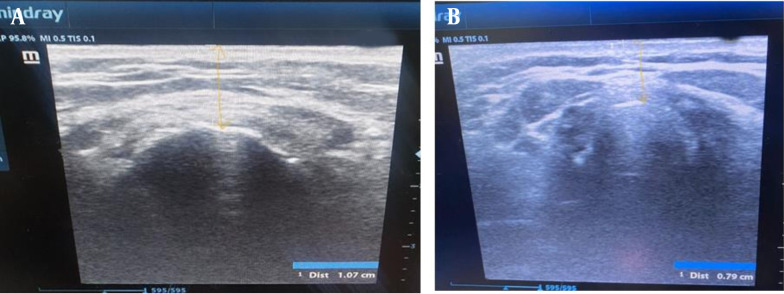
The anterior neck soft tissue thickness at A, the level of the hyoid bone and B, the vocal cords (yellow arrow).

The US device was Mindray diagnostic US system (China) model Z60. Linear and curved probes were used. The hyomental distance of the patient in the neutral position of the neck and in the fully extended neck, calculating the ratio between both of them, were shown using the curved probe (Figure 1). The anterior neck soft tissue thickness at the level of the hyoid bone (ANS-Hyoid) and vocal cords (ANS-VC) were shown using the linear probe (Figure 2). 

**Figure 3.**
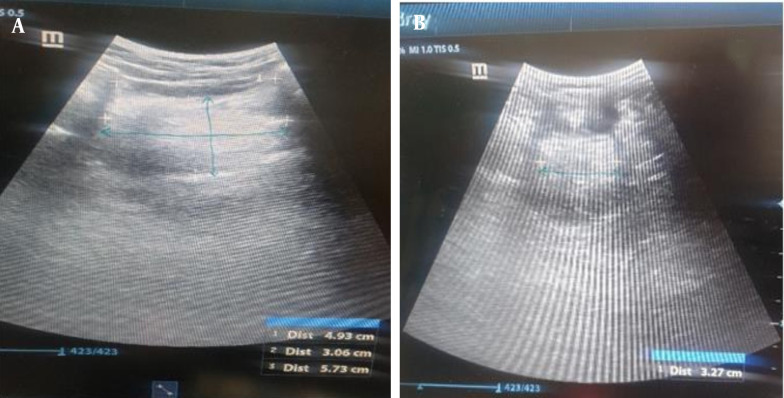
A, Midsagittal cross-sectional area of the tongue and B, the width of the tongue obtained from transverse sonograms (blue arrow).

Tongue volume was derived from the multiplication of the midsagittal cross-sectional area of the tongue by its width obtained from transverse sonograms using the curved probe (Figure 3). 

The conventional clinical airway evaluation methods involved modified Mallampati classification, the distance of thyromental, inter-incisor gap, neck mobility, and sternomental distance. 

Each separate finding by the US and conventional clinical airway assessment methods before anesthesia induction correlated to the CLG of the same patient after the induction of anesthesia. The CLG is classified under four grades. Grade I is a full view of the glottis. Grade II is a partial view of the glottis or arytenoids. Grade III is only epiglottis seen. Grade IV is neither glottis nor epiglottis visible. Grades I and II are categorized as easy laryngoscopy. Grades III and IV are categorized as difficult laryngoscopy (8). 

Oxygen saturation (SpO_2_), electrocardiogram (ECG), non-invasive blood pressure measurement, and end-tidal CO₂ were monitored. Fentanyl 1 ug/kg, propofol 2 mg/kg, and succinylcholine 1.5 mg/kg had been given following preoxygenation. Anesthesia was preserved by isoflurane minimum anesthetic concentration (MAC) of 1.15% and atracurium (loading dose 0.5 mg/kg, then 0.1 mg/kg increments every 20-30 minutes). At the end of the surgery, isoflurane was stopped, and the process of reversing muscular relaxing was initiated using neostigmine 0.05 mg/kg, and atropine 0.01 mg/kg was done following the recipient's return of spontaneous respiration. 

### 3.1. Sample Size Calculation

Assuming that the total number of obese individuals hospitalized in the operation room is 100 cases per year and the positive predictive value of US in the prediction of CLG is 33.3% (9), the total sample size was 78 cases using OpenEpi, Power of the test 80%, and 95% confidence interval (CI).

### 3.2. Statistical Analysis

The statistical analysis was conducted using SPSS v26 software (IBM Inc., Chicago, IL, USA). The normality of the data distribution was assessed using the Shapiro-Wilks test and histograms. The mean and standard deviation (SD) of quantitative parametric factors were reported and contrasted among both categories using an unpaired Student's t-test. The quantitative non-parametric variables were evaluated using the Mann-Whitney test. The qualitative parameters were evaluated utilizing either the Chi-square test or Fisher's exact test. The area under the receiver operating characteristic curve (ROC) curve denotes the diagnostic performance of the test. Spearman correlation was performed between US measures and classical measures to CLG. A p-value < 0.05 was considered statistically significant.

## 4. Results

**Figure 4.**
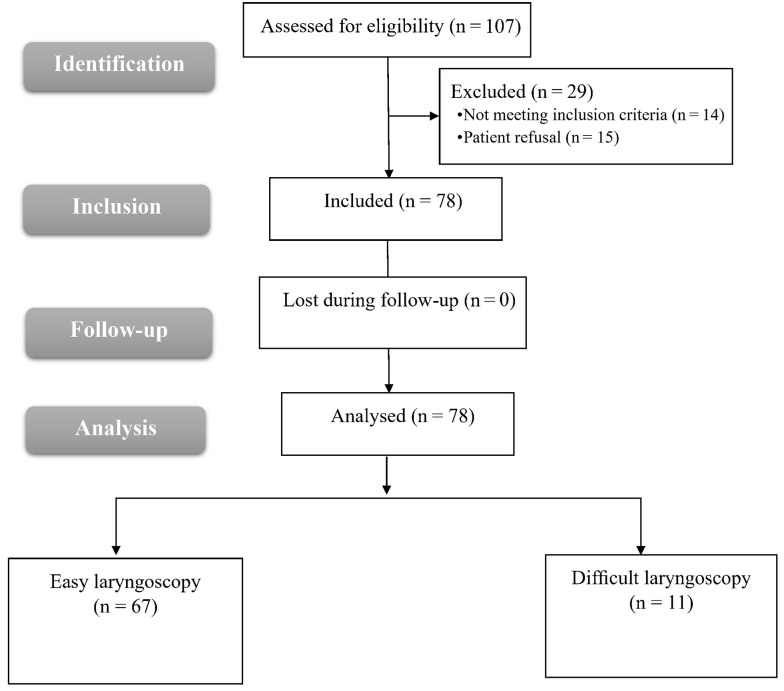
Flowchart of the enrolled patients

In this study, 107 patients were assessed for eligibility, 14 patients did not meet the criteria, and 15 patients refused to participate in the study. The remaining 78 patients were divided into two groups: Group 1, easy laryngoscopy CLG I and II (n = 67), and group 2, difﬁcult laryngoscopy CLG III (n = 11). All allocated patients were followed up and analyzed statistically (Figure 4). 

No statistically substantial variation was observed among both categories regarding weight, age, BMI, skin to the hyoid bone, pre-epiglottis space (Pre-E), tongue volume, transverse scan of the tongue’ width, and midsagittal cross-sectional area of the tongue. A highly statistically substantial variation was observed among the two categories regarding the US measures duration, skin to the anterior commissure, the distance between the epiglottis and a point midway the vocal cords, the ratios of Pre-E over the distance between the epiglottis, and a point midway vocal cords and hyomental distance neutral. A statistically substantial variation existed among both categories regarding hyomental distance extension and the ratio of hyomental distance extension/neutral (Table 1). 

**Table 1.** Demographic Data and Ultrasound Measurements Among Patients with Different Cormack and Lehane Grades

Variables	Easy Laryngoscopy CLG I and II (n = 67)	Difﬁcult Laryngoscopy CLG III (n = 11)	U	P
**Age (y)**	37.72 ± 8.54	36.45 ± 6.93	345.5	0.740
**Height (cm)**	157.19 ± 4.57	165.7 ± 8.88	126.0^a^	< 0.001^a^
**Weight (kg)**	106.0 ± 12.30	110.0 ± 12.04	273.0	0.159
**BMI (kg/m** ^**2**^ **)**	42.34 ± 4.18	40.64 ± 8.23	330.0	0.577
**Duration of ultrasound measures (min)**	13.34 ± 0.83	17.0 ± 1.0	0.000^a^	< 0.001^a^
**Skin to anterior commissure (cm)**	0.74 ± 0.10	0.40 ± 0.15	17.500^a^	< 0.001^a^
**Skin to hyoid bone (cm)**	0.94 ± 0.22	1.02 ± 0.10	299.0	0.315
**Pre-epiglottis space (cm)**	1.52 ± 0.53	1.70 ± 0.12	249.0	0.085
**Distance between the epiglottis and a point midway the vocal cords (cm)**	0.98 ± 0.38	0.60 ± 0.04	126.0	< 0.001^a^
**The ratio of Pre-E over the distance between the epiglottis and a point midway the vocal cords**	1.60 ± 0.42	2.85 ± 0.18	0.000^a^	< 0.001^a^
**Tongue volume (cm** ^**3**^ **)**	127.7 ± 40.47	126.0 ± 23.85	359.0	0.891
**Transverse scan width (cm)**	4.32 ± 0.65	4.06 ± 0.83	317.0	0.457
**The midsagittal cross-sectional area of the tongue (cm** ^**2**^ **)**	29.01 ± 7.72	32.04 ± 8.80	261.0	0.121
**Hyomental distance neutral (cm)**	4.84 ± 0.57	4.29 ± 0.38	160.0^a^	0.003^a^
**Hyomental distance extension (cm)**	5.27 ± 0.51	4.86 ± 0.25	193.0^a^	0.011^a^
**The ratio of hyomental distance extension/neutral**	1.09 ± 0.06	1.13 ± 0.04	197.50^a^	0.013^a^

Abbreviations: CLG, Cormack-Lehane grading; IQR, interquartile range; SD, standard deviation; U, Mann-Whitney test; P, P-value for comparing between the two studied categories.

^a^ Statistically significant at P ≤ 0.05.

A highly substantial variation existed among both categories regarding Mallampati. No statistically substantial variation existed among both categories regarding sternomental and thyromental distance (Table 2). 

**Table 2.** Classical Measurements Among Patients with Different Cormack and Lehane Grades ^a^

Variables	No.	Easy Laryngoscopy CLG I and II (n = 67)	Difﬁcult laryngoscopy CLG III (n = 11)	χ^2^	P
**Sternomental distance (cm)**	11	7 (10.4)	4 (36.4)	4.687	^MC ^p = 0.077
	12	53 (79.1)	7 (63.6)		
	13	7 (10.4)	0 (0.0)		
**Thyromental distance (cm)**	5	7 (10.4)	4 (36.4)	4.687	^MC ^p = 0.077
	6	53 (79.1)	7 (63.6)		
	7	7 (10.4)	0 (0.0)		
**Mallampati**	2	53 (79. 1)	0 (0.0)	27.149	^FE ^p < 0.001^b^
	3	14 (20.9)	11 (100.0)		

Abbreviations: CLG, Cormack-Lehane grading; χ^2^: Chi-square test; MC, Monte Carlo; FE, Fisher’s exact; P, P-value for comparing between the two studied categories.

^a^ Data are expressed as number (percentage) unless otherwise indicated.

^b^ Statistically significant at P ≤ 0.05.

A high statistically substantial positive association existed among CLG and duration of US measures, the ratio of Pre-E to the distance between epiglottis and a point midway vocal cords, pre-epiglottic space, ratio of hyomental distance extension/hyomental distance neutral, and Mallampati; however, there was a significant negative correlation with skin to anterior commissure, hyomental distance extension, hyomental distance neutral, sternomental distance, and thyromental distance (Table 3). 

**Table 3.** Correlation Between Ultrasound Measurements and Classical Measurements to Cormack-Lehane Grades

Variables	r ^a^	P
**Duration of ultrasound measures**	0.83	< 0.001^b^
**Skin to anterior commissure**	-0.66	< 0.001 ^b^
**Skin to hyoid bone**	0.04	0.67
**Distance between epiglottis and a point midway the vocal cords**	-0.2	0.24
**Pre-epiglottic space**	0.42	< 0.001 ^b^
**The ratio of Pre-E over the distance between the epiglottis and a point midway the vocal cords**	0.85	< 0.001 ^b^
**Tongue volume**	-0.05	0.62
**Transverse scan width**	0.13	0.22
**Midsagittal cross-sectional area of the tongue**	-0.19	0.08
**Hyomental distance neutral**	-0.55	< 0.001 ^b^
**Hyomental distance extension**	-0.54	< 0.001 ^b^
**The ratio of hyomental distance extension/hyomental distance neutral**	0.26	0.01 ^b^
**Sternomental distance**	-0.6	< 0.001 ^b^
**Thyromental distance**	-0.6	< 0.001 ^b^
**Modified Mallampati Score**	0.55	< 0.001 ^b^

^a^ r, correlation coefficient.

^b^Statistically significant at P ≤ 0.05.

**Figure 5.**
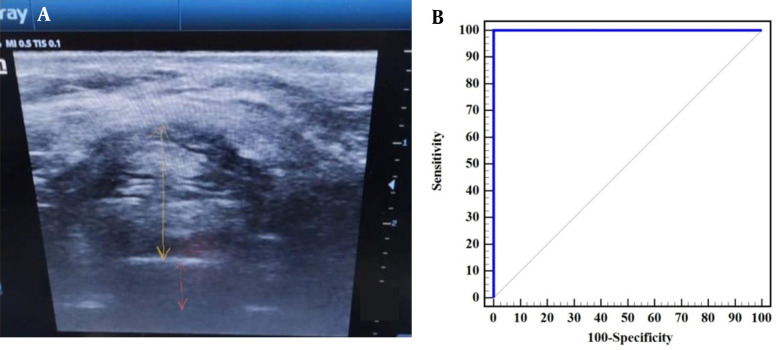
A, the preepiglottic space (yellow arrow) and distance between the epiglottis and a point between the vocal cords (orange arrow), B, ROC curve to predict the ability of ratio of preepiglottic space over the distance between the epiglottis and a point midway vocal cords to differentiate between grade III and grade (I and II) of Cormack-Lehane.

The ratio between Pre-E over the distance between the epiglottis and a point midway the vocal cords can discriminate between CLG III vs. grade I and II with sensitivity 100% and specificity 100% and area under the curve (AUC) 1 at cut off > 2.23 (Table 4 and Figure 5). 

**Table 4.** Power of Ultrasound Measures and Classical Measures in Discriminating Between Cormack and Lehane Grades (Grade III vs. Grade I and II)

Test Result Variable(s)	AUC	P-Value	Cut off	No. (%)	
				Sensitivity	Specificity
**Skin to hyoid bone**	0.594	0.318	> 1.07	54.55	79.10
**Skin to anterior commissure**	0.976	< 0.001 ^a^	≤ 0.55	100	89.55
**Distance between epiglottis and a point midway the vocal cords**	0.829	< 0.001 ^a^	≤ 0.62	90.91	79.10
**Pre-epiglottic space**	0.662	0.0860	> 1.59	90.91	68.66
**The ratio of Pre-E over the distance between the epiglottis and a point midway vocal cords**	1.000	< 0.001^a^	> 2.23	100	100
**Tongue volume**	0.513	0.892	≤ 140	100	35.82
**Transverse scan width of the tongue**	0.570	0.460	≤ 3.61	63.64	79.10
**Midsagittal cross-sectional area of the tongue**	0.646	0.123	> 39.72	45.45	98.51
**Hyomental distance neutral**	0.783	0.003 ^a^	≤ 3.96	54.55	100
**Hyomental distance extension**	0.738	0.012 ^a^	≤ 5.12	100	56.72
**The ratio of hyomental distance extension/hyomental distance neutral**	0.732	0.014 ^a^	> 1.07	100	53.73
**Sternomental distance**	0.43	0.55	12.5	100	10
**Thyromental distance**	0.410	0.525	5.5	80	10
**Modified Mallampati**	0.8	0.05	2-3	100	80

Abbreviations: AUC, the area under the curve; P, the P-value for comparing between the two studied categories.

^a^ Statistically significant at P ≤ 0.05.

## 5. Discussion

The requirement to effectively manage the airway is prevalent among professionals in the fields of anesthesia, respiratory care, critical services, and emergency medicine. Several prognosticators have been proposed to assist in identifying the potentially dangerous 'can't ventilate, can't intubate' situation. This catastrophic outcome might manifest in 1 out of every 1 000 elective instances and 1 out of every 250 fast sequence instances (10).

In this work, a highly substantial variation existed among the studied categories (easy laryngoscopy and difficult laryngoscopy) regarding the time of US measures and skin to the anterior commissure. No substantial variation existed among the studied categories regarding skin to hyoid bone. The findings of the present study are not supported by the study of Sotoodehnia et al. (11). They stated that distance from skin to hyoid bone (DSHB) had a significant relationship with challenging laryngoscopy and demonstrated that the distance from skin to vocal cords (DSVC) had a substantial association with challenging laryngoscopy.

The results of the present study are in line with the work of Abdelhady et al. (12), who stated that patients with difficult laryngoscopy demonstrated substantially larger thickness of distance from skin to epiglottis (DSE). Another study by Gupta et al. (13) proved that as the Pre-E became bigger and the distance between the epiglottis to vocal cords shrank, the CLG would be that of the difficult laryngeal visualization. However, if the Pre-E measurement was large while the ratio was also found to be large due to long epiglottis to vocal cord distance, it would result in a CLG of easy laryngeal visualization. However, in the study of Yadav et al. (14), the ratio of Pre-E (Pre-E)/epiglottis to vocal cords (E-VC) was substantially greater among individuals with challenging intubation.

In contrast to the results of the present study, the work of Yadav et al. (14) reported that the hyomental distance ratio substantially decreased among individuals with challenging intubation. Huh et al. (15) demonstrated that the HMDR alone had the greatest accuracy in predicting difficulty laryngoscope. The best threshold for predicting a challenging airway was determined to be 1.2, which is not in agreement with the findings of the present study. 

The present work demonstrated that no substantial variation existed among the studied categories (easy laryngoscopy and difficult laryngoscopy) regarding the tongue volume, transverse scan width, and midsagittal cross-sectional area of the tongue. Wojtczak (16) showed that the results were the same as those obtained in the current study, as they declared the tongue volume had no role in detecting a difficult laryngoscopy scenario, explaining that it could be due to the importance of measuring it in relation to the mandibular volume. 

In the current study, the ratio between the tongue volume to the mandibular volume was not estimated. A study conducted by Hui and Tsui et al. (17) demonstrated that the utilization of ultrasonography alone is enough to determine a case of potentially challenging intubation prior to applying the laryngoscopy. 

Moreover, in the present work, the thickness of the tongue was utilized in combination with a variety of other variables assessed by both US and clinical assessment methods to forecast a case of difficult intubation preoperatively and was not used as the sole parameter. In addition, in the work performed by Hui and Tsui et al. (17), the patient’s weight was not taken into consideration in the studied subjects, unlike the current study where the study subjects were obese with a BMI of 30 to < 30 kg/m².

The findings of the present study are supported by the study of Yadav et al. (14), who reported that a statistically substantial difference was noted in modified Mallampati. Additionally, Abdelhady et al. (12) showed that during the evaluation of pre-intubation screening examinations, substantial disparities were observed in the Mallampati score. Brodsky et al. (18) showed that the clinical assessment points, including the distance of thyromental, the opening of the mouth, mobility of the neck, and modified Mallampati score, were useless as predictors of difficult airway which are not in line with the present work.

In agreement with the present study, Gupta et al. (13) stated a strong negative association between the distance between the epiglottis and the vocal cords (E-VC) with the CLG.

Regarding diagnostic performance for the ratio of Pre-E over the distance between the epiglottis and a point midway vocal cords to discriminate difﬁcult laryngoscopy, Gupta et al. (13) are in agreement with the results of the present study as they stated that the ratio of Pre-E and E-VC distances (Pre-E/E-VC) might be used to predict Cormack-Lehane grades. Based on this set of standards, the ability to anticipate challenging intubation (Pre-E/E-VC 2-3) shows a great level of specificity but a very poor level of sensitivity, in contrast with the findings of Soltani Mohammadi et al. (19), who reported modest relationships between the Pre-E and CLG I, II, and III. In contrast to the results of Andruszkiewicz et al. (20) who demonstrated that hyomental distance in extension had the greatest AUC value of all the evaluated indicators of challenging laryngoscope. The results of the present study are in contrast with Abdelhady et al.’ results (12) that revealed a good association between US measurements of DSE and CLG in the Egyptian population; therefore, it might be seen as an indicator of challenging laryngoscopy with high sensitivity and specificity.

The present work has numerous limitations, which are the limited sample size, the exclusive focus on a single race in the US measures, conducted by a sole investigator, which might introduce potential bias, and No CLG IV cases. It is recommended to use rocuronium instead of succinylcholine due to the side effects of succinylcholine.

### 5.1. Conclusions

The sonographic assessment of the upper airway aids in predicting individuals who are likely to have a challenging airway. The sonographic variable ratio of Pre-E to the distance between the point midway vocal cords and epiglottis is a reliable indicator of challenging laryngoscopy.

## Data Availability

No new data were created or analyzed in this study. Data sharing does not apply to this article.

## Clinical Trial Registration Code:

NCT04205331.
